# Unpacking the Relationship between Sexual and Gender Minority Adverse Childhood Experiences and Psychological Distress: the Mediating Role of Self-Compassion and Fear of Compassion from others

**DOI:** 10.1007/s40653-026-00879-2

**Published:** 2026-04-24

**Authors:** Han Chen, Eddie S. K. Chong, Randolph C. H. Chan

**Affiliations:** 1 Department of Social Work and Social Administration, The University of Hong Kong, Hong Kong, China; 2 Centre on Behavioral Health, The University of Hong Kong, Hong Kong, China; 3 Department of Social Work, The Chinese University of Hong Kong, Hong Kong, China

**Keywords:** SGM-ACEs, Self-compassion, Fear of compassion from others, Mental health, Trauma

## Abstract

Sexual and gender minority individuals experience higher rates of adverse childhood experiences (ACEs) than heterosexual individuals. Yet, the general ACE framework ignores unique ACEs faced by SGM individuals (i.e., cisheterosexism during childhood). The Sexual and Gender Minority ACE (SGM-ACE; Schnarrs et al., 2022, 2023) framework fills this gap by accounting for ACEs that are unique for SGM individuals resulting from systems of oppression (i.e., cisheterosexism during childhood). However, past studies employing this framework were conducted in the U.S. context, limiting the understanding of the prevalence of SGM-ACEs and their associations with psychological outcomes in other contexts such as Hong Kong where LGBTQ+ policies and laws are still progressing. Furthermore, prior research has rarely explored how SGM-ACEs adversely affect psychological outcomes through impeding capacities for compassion of SGM individuals. Therefore, utilizing data from 736 SGM individuals in Hong Kong, this study addressed these important gaps by exploring the prevalence of SGM-ACEs and examined the association between SGM-ACEs and psychological distress among Hong Kong SGM individuals. Moreover, self-compassion and fear of receiving compassion from others were tested as mediators of the effects of SGM-ACEs on psychological distress. Results show that a large proportion of SGM individuals in Hong Kong have experienced SGM-ACEs (89.3%) and these traumatic childhood experiences were significantly linked to higher psychological distress in adulthood. Both self-compassion and fear of compassion from others significantly mediated this relationship. This study demonstrates the significance of considering ACEs specifically related to SGM identities. Findings offer critical insights into practice, clinical treatment, psychoeducation, and policies.

Adverse childhood experiences (ACEs) refer to traumatic and stressful experiences occurring prior to adulthood and are traditionally considered to include abuse, neglect and other household challenges (Higgins et al., 2023). The high global prevalence of ACEs demonstrates a major concern due to their deleterious effects on child development and mental health issues that extend into adulthood (Stoltenborgh et al., 2015). Sexual and gender minority (SGM) individuals even report higher rates of ACEs compared to their heterosexual counterparts (Austin et al., 2016; Schnarrs et al., 2022). However, most past studies focusing on ACEs of SGM people have utilized a general framework of ACEs and thus overlooked specific ACEs related to their marginalized identities (Schneeberger et al., 2014). An emerging sexual and gender minority adverse childhood experience (SGM-ACE) framework appears to fill this knowledge gap (Schnarrs et al., 2022, 2023) as it more precisely captures the unique early life adversities encountered by SGM individuals by conceptualizing SGM identity-related discrimination and stigma in childhood as forms of SGM-specific ACEs. Although a handful of studies in the U.S. have employed this framework and revealed the detrimental effect of SGM-ACE on mental health in later life, no study has examined this relationship in other countries or regions such as Hong Kong where SGM people face different structural challenges. Furthermore, few studies have investigated the underlying mechanism of this negative link between SGM-ACE and mental health and evidence was limited. Only one study has identified perceived family support as a significant mediator in the link between SGM-ACE and anxiety, among other sources of support and outcomes (Dorri et al., 2023). However, since childhood adversity impacts not only individuals’ perceived access to support but also the way people relate to themselves and others (Dorri et al., 2023; Guo et al., 2021; Matos et al., 2013), the ability to relate to oneself in a kind way and receive compassion from others might help explain the association between SGM-ACEs and psychological distress among SGM people. Addressing this knowledge gap not only contributes to ACE-related research and theories, but also informs intervention and training efforts. Hence, the present study aimed to examine the association between SGM-ACEs and psychological distress, with a particular focus on the mediating roles of self-compassion and fear of compassion from others among SGM individuals in Hong Kong.

## SGM-ACEs and Mental Health Outcomes

When studying ACEs pertaining to SGM individuals, the traditional ACE framework may lack consideration of unique and specific ACEs faced by these marginalized populations that result from systems of power and oppression (Bernard et al., 2021; Schnarrs et al., 2022). Schnarrs et al. (2022, 2023) resolved this problem by conceptualizing cisheterosexist experiences during childhood as specific forms of ACEs for SGM people. Cisheterosexism privileges heterosexuality and cisgender identity (i.e., consistency of sex assigned at birth and gender identity) while devaluing diverse non-heterosexual, non-cisgender identities and nonconforming gender expressions (Schnarrs et al., 2022). As such, unlike the traditional ACE framework focusing primarily on general childhood maltreatment from caregivers (e.g., emotional and physical abuse and neglect, sexual abuse) or household dysfunction such as divorce or domestic violence, the SGM-ACE framework emphasizes victimization or harsh treatment within families due to family members’ devaluing attitudes toward stigmatized identities of SGM individuals. Moreover, Schnarrs et al. (2022, 2023) expanded the SGM-ACE framework beyond familial adversities to include social and structural challenges faced by SGM individuals, such as school bullying, prejudice from religious communities and institutionalization. Institutionalization refers to childhood experiences in foster care, juvenile detention, or a mental hospital. For example, SGM youth may be placed in foster care following events that compromised their safety, such as abuse by adults due to their sexual orientation and/or gender identity or expression. However, only a limited number of studies has utilized the SGM-ACE framework when examining its contribution to mental health outcomes and revealed a positive relationship between SGM-ACEs and psychological distress (Dorri et al., 2023; Schnarrs et al., 2022, 2023). Notably, these findings were all based on U.S. samples. Therefore, the prevalence of SGM-ACEs, as well as its long-lasting effects on psychological distress in adulthood remain unclear in other countries or regions with different sociopolitical environment, such as Hong Kong.

Hong Kong has been strongly influenced by both British colonial history and traditional Chinese culture. Under this context, SGM individuals are at great risk of experiencing discrimination across multiple contexts, such as families, schools and religious organizations due to Christian beliefs and Confucian family values (Suen & Wong, 2017). Christianity, introduced during British colonization, has mostly regarded homosexuality as sinful, reinforcing stigmatizing attitudes toward SGM individuals in Hong Kong (Chan, 2005). Meanwhile, Confucian ideals emphasizing filial piety and continuation of the family lineage have also reinforced heteronormative expectations and exerted significant pressure for SGM individuals to conform to family norms. However, anti-discrimination laws for protecting SGM individuals or legal recognition of same-sex relationships are lacking in Hong Kong (Wong et al., 2025). Despite these challenges, it is noteworthy that public attitudes have shifted in a positive direction in recent years. For instance, comparisons across three adolescent cohorts indicate that attitudes toward same-sex relationships became increasingly inclusive from 2011 to 2021 (Li et al., 2025). Support for same-sex marriage also improved from 38% in 2013 to 60% in 2023 (Wong et al., 2025). Considering the dearth of studies investigating SGM-ACEs in the Hong Kong context, this study filled this critical research gap.

### SGM-ACEs and Compassion

SGM-ACEs might thwart sexual and gender minority people’s capacities for compassion, including generating compassion for oneself and receiving compassion from others. Correspondingly, there are two key constructs. The first one is self-compassion, which involves showing care, kindness, and non-judgment toward oneself, especially during hard times or suffering (Neff, 2003). The second is fear of compassion from others, referring to fears and difficulties in experiencing and receiving compassion from others (Gilbert et al., 2011). Although fewer studies on ACEs have focused on fear of compassion compared to self-compassion, it is vital to pay attention to this construct because it involves an active resistance to compassionate experiences that is distinct from the mere absence of compassion, and such fearful reactions can impede individuals’ motivations and abilities to reap benefits from compassionate behaviors (Gilbert et al., 2011).

Attachment theory provides theoretical support for the negative effect of SGM-ACEs on self-compassion and fear of compassion from others. Specifically, attachment theory posits that early interactions with significant others (e.g., parents and peers) significantly shape the development of internal working models, which serve as cognitive and relational templates regarding the self and others (Bowlby, 1973). Early interpersonal experiences characterized by responsiveness and care foster positive working models of the self as valued and worthy of care and of others as safe and reliable (Bowlby, 1973; Raque et al., 2023). However, for SGM people experiencing adverse childhood experiences including familial rejection, school bullying, religious trauma and institutionalization due to their devalued identities, there is a scarcity of availability, care and affection in their interpersonal relationships, which are essential components for building a positive internal working model (Bretherton, 1985). Rather, these traumatic identity-related childhood experiences can accumulate to result in the formation of negative internal working models and disrupt their attachment security (Skidmore et al., 2023).

Due to repeated exposure to discrimination and victimization, individuals with SGM-ACEs tend to hold a negative working model of themselves as unvalued and unworthy of love and compassion, suppressing their ability to recognize and acknowledge their needs for care (Zhao et al., 2023; Skidmore et al., 2023). Meanwhile, these childhood oppressive interpersonal experiences hinder SGM individuals from establishing a compassionate relational template, thus impeding their ability to develop a caring self-to-self relating (Lathren, 2023). Furthermore, SGM-ACEs give rise to a negative working model of others as untrustworthy, unavailable and condemning (Matos et al., 2013). Since trustworthy relationship bonds are prerequisites for accepting affiliation and support from others, receiving compassion from others could thus become fearful and frightening when security and trust are absent in self-other relating (Kelly et al., 2012). The fear experienced by SGM individuals when receiving compassion from others may reflect a protective adaptation to early experiences in which they faced systematic victimization or punishment related to their minority identities (Steindl et al., 2023). This defensive mechanism manifests in insecure attachment, involving either avoidant strategies that minimize potential harm and hostility through emotional distancing, or anxious strategies characterized by preoccupation with rejection and abandonment stemming from internalized expectations of stigma (Collins, 1996; Diamond & Alley, 2022).

### The Mediating Role of Compassion

Robust empirical studies show the positive effects of self-compassion on mental health outcomes across different populations, including sexual and gender minority people (Carvalho & Guiomar, 2022; MacBeth & Gumley, 2012). Conversely, fear of compassion from others contributes to elevated psychological distress and worse psychological functioning (Gilbert et al., 2011; Kirby et al., 2019). As a result, the attachment theory and empirical evidence collectively indicate self-compassion and fear of compassion from others as potential mediators of the link between SGM-ACEs and psychological distress.

Although previous empirical studies have found significant mediating effects of self-compassion on the relationship between ACEs and psychological outcomes in general populations such as adolescents and college students (Guo et al., 2021; Kwok et al., 2022), limited research has utilized the SGM-ACE framework and examined the mediating role of self-compassion among SGM people. In addition, although a handful of studies have explored the mediating effect of fear of compassionate experiences, they have primarily focused on fear of self-compassion while rarely considering fear of compassion from others (Boykin et al., 2018; Messman-Moore & Bhuptani, 2020). This gap is noteworthy, as individuals with limited knowledge about SGM issues, including therapists, may question the effectiveness of their support and affiliations when working with SGM people. To date, only one study has revealed that fear of compassion from others could mediate the association between shame traumatic memory and psychological distress in a general community sample (Matos et al., 2017), providing partial support for the mediating role of fear of compassion from others in the present study.

### The Present Study

The present study aimed to investigate the prevalence of SGM-ACEs among SGM people in Hong Kong and examine the association between SGM-ACEs and psychological distress (i.e., depression and anxiety). We also explored whether self-compassion and fear of compassion from others could serve as underlying mediating mechanisms. Based on theory and prior empirical findings, we hypothesized that SGM-ACEs would be associated with higher psychological distress and both self-compassion and fear of compassion from others would be significant mediators of this link. Specifically, SGM-ACEs would be related to lower self-compassion and higher fear of compassion from others, which in turn would be linked to higher psychological distress.

## Method

### Participants and Procedure

This study conducted a secondary analysis of data from a larger project titled *LGBTQ Community Survey*, an online survey that takes approximately 30 min to complete on Qualtrics. Participants were recruited from September to November in 2023 through advertisements on Facebook and Instagram, as well as mass emails through the University of Hong Kong. Interested individuals first completed a brief eligibility screening. Those who are 16 years or older, self-identified as SGMs, and currently living in Hong Kong were then directed to the consent form and, upon providing consent, proceeded to the survey. After completion, participants received HKD 50 cash as a token of appreciation for their time. The project was approved by the Human Research Ethics Committee of the University of Hong Kong (EA220083).

Responses that were bots-generated or without consent form were first excluded, resulting in 1505 remaining participants. To ensure the validity and quality of the online survey responses, we further excluded responses that met any of the following criteria: (a) incorrect responses to one or both validity check items (e.g., “Please select ‘strongly agree’ for this item”) (259 participants); (b) completion of the entire survey in less than 15 min, which was below the minimum plausible duration based on all measures included in the survey including those not reported in the present study (84 participants); (c) endorsement of a seriousness rating of 3 or below on a 0-7 scale assessing survey engagement (“How seriously did you take the survey?”) (29 participants); (d) duplicate email addresses, for which only the first submission was retained (resulting in the exclusion of five responses); and (e) submissions flagged by Qualtrics’ data-quality checks as requiring further review, which then did not pass manual verification, including review of open-ended responses and email address validation (15 responses). Consequently, the final analytic sample consisted of 1,113 participants. Since the primary aim of this study is to understand SGM-ACEs among Hong Kong people and the effect of SGM-ACEs on later outcomes in adulthood, we limited our sample to SGM-identifying individuals who (a) spent a significant amount of time in Hong Kong during childhood (before the age of 18), (b) could comprehend Chinese and (c) were 18 years or older. The final analytic sample consisted of 736 SGM people in Hong Kong (*M*_age_ = 26.07, *SD*_age_ = 8.02). Table 1 shows their demographic information.

**Table 1:** Demographics of study sample (*N* = 736)

	*n*	%
Gender		
Cisgender Men	308	41.9
Cisgender Women	335	45.5
Transgender/gender queer/gender non-binary/gender fluid	93	12.6
Sexual orientation		
Gay/Lesbian	402	54.6
Bisexual	260	35.3
Asexual/gray-A/demisexual	30	4.1
Queer	44	6.0
Educational level		
Junior high to high school	130	17.7
Associate degree or Higher diploma	81	11.0
Bachelor’s degree	384	52.2
Postgraduate degree	139	18.9
Income		
Receiving Comprehensive Social Security Assistance	8	1.1
Less than HKD 5,000	261	35.5
HKD 5,001–30,000	303	41.2
HKD 30,001–50,000	89	12.1
HKD 50,001–70,000	39	5.3
More than HKD 70,001	26	3.5
Religion/Spirituality		
No religion	528	71.7
Atheism	26	3.5
Agnosticism	19	2.6
Buddhism	36	4.9
Taoism	9	1.2
Christianity	83	11.3
Catholicism	24	3.3
Other religious/spiritual affiliations	11	1.5

(1) The total percentage by adding up different categories of educational level and monthly income was less than 100% due to missing data

(2) Monthly income was assessed with 21 options and we grouped these categories into broader categories in Table 1 to simplify the interpretation of the income distribution of our participants. The average monthly salary in Hong Kong across different industries in 2025 was HKD 19,565 (Census and Statistics Department, 2025). The poverty line (i.e., the income threshold to define poor households and their population) in Hong Kong was around HKD 5,000 (Oxfam Hong Kong, 2024)

(3) Participants indicated their educational level from 1 = “elementary school or below” to 10 = “doctorate degree” and some categories were combined in Table 1 for simplicity

### Measures

#### Sexual and Gender Minority Adverse Childhood Experiences

The 8-item Sexual and Gender Minority Adverse Childhood Experiences Scale (SGM-ACEs; Schnarrs et al., 2023) was used to evaluate the frequency of cisherterosexist experiences before 18 years old. Specific items could be found in Table 2. Participants were asked to provide responses based on a five-point Likert scale from 0 (*never*) to 4 (*always*). Higher average scores of this scale represent higher exposure to SGM-ACEs. The internal consistency of this scale was acceptable, Cronbach’s α = 0.72.

**Table 2:** Frequency of Reported Exposure to SGM-ACEs (*N* = 736)

SGM-ACE item	Any exposure	Never	Seldom	Sometimes	Very often	Always
You were bullied in school by other children, teachers, staff, or school administrators (i.e., principal) because of your sexuality or gender identity.	30.3%	69.7%	17.5%	8.2%	3.0%	1.6%
You saw/heard of other LGBTQ+ people being physically harmed.	62.8%	37.2%	29.8%	27.4%	4.8%	0.8%
You were in foster care, juvenile detention, or a mental hospital.	3.8%	96.2%	0.6%	0.6%	0.4%	0.1%
Family members said transphobic, homophobic, or biphobic things about you or other people on a regular basis in person or on social media.	61.5%	38.5%	24.4%	24.7%	8.3%	4.1%
Religious leaders at your church or other faith community said homophobic and transphobic things, such as teaching that the Bible or other texts condemn homosexuality or transgenderism.	44.2%	55.8%	12.4%	16.8%	10.2%	4.8%
You were punished, shamed, or yelled at by family members for not conforming to gender expectations (being too much or not manly enough, being too feminine or not feminine enough).	41.3%	58.7%	19.9%	13.7%	5.0%	2.7%
You felt pressure to have sex or relationships that you did not want to protect your family from discovering your gender or sexuality.	26.9%	73.1%	11.1%	8.0%	4.5%	3.3%
You were kicked out of your home or ran away because you were LGBTQ+.	6.1%	93.9%	2.3%	2.6%	0.7%	0.5%

#### Self-compassion

Self-compassion was measured by the 20-item Sussex-Oxford Compassion for the Self Scale (Gu et al., 2020). Participants responded to items (e.g., “when bad things happen to me, I feel caring towards myself”) based on a 5-point Likert scale from 1 (*not at all true*) to 5 (*always true*). Higher average scores of all items indicate higher self-compassion. The internal reliability of this scale in our sample was excellent, Cronbach’s α = 0.90.

#### Fear of Compassion from others

This was measured by fear of compassion from others scale from Fears of Compassion Scales (Gilbert et al., 2011). The original scale consists of 13 items, and we selected 6 items with highest factor loadings from Gilbert’s study (2011) to reduce the length of our questionnaire. Sample item used was “feelings of kindness from others are somehow frightening”. Participants responded on a five-point Likert scale ranging from 0 (*don’t agree at all*) to 4 (*completely agree*). Higher mean scores of these 6 items represent higher levels of fear of compassion from others. The internal reliability in the current sample was good, Cronbach’s α = 0.87.

#### Psychological Distress

Psychological distress was indicated by depression and anxiety. Depression was assessed using the Patient Health Questionnaire-2 (PHQ-2; Kroenke et al., 2003) and anxiety was measured by Generalized Anxiety Disorder-2 scale (GAD*-*2; Kroenke et al., 2007). Participants rated both scales based on a four-point Likert scale ranging from 0 (*not at all*) to 3 (*nearly every day*). Higher average scores of corresponding items for PHQ-2 and GAD-2 indicate higher levels of depression and anxiety. The internal consistencies of PHQ-2 and GAD-2 were acceptable and good (Cronbach’s α = 0.79 and 0.83, respectively).

#### Covariates

Covariates included age, gender, sexual orientation, educational level and income. Participants were asked to report their educational level ranging from 1 = elementary school or below to 10 = doctorate degree. Monthly income was assessed by providing 21 income options, with categories increasing in HKD 5,000 increments, namely, 1 = I currently receive Comprehensive Social Security Assistance (CSSA), 2 = Below $5,000, 3 = $5,001-$10,000 … 20 = $95,001-$100,000 and 21 = $100,001 or above. As a result, age, educational level and income were all treated as continuous variables. In terms of gender, we first asked participants whether they identified as transgender. Then we asked their specific gender identities with four options: (a) female, (b) male, (c) gender queer or gender non-binary or gender fluid and (d) other gender identities. We further created a dichotomous variable for subsequent analyses based on responses to these two items assessing gender identity. For participants who were cisgender male or female, they were coded as cisgender (value = 0) whereas participants who identified as transgender, gender queer, gender non-binary or gender fluid were coded as gender diverse (value = 1). Regarding sexual orientation, SGM individuals were asked to select their responses from four options: (a) gay or lesbian, (b) bisexual, (c) asexual or gray-A or demisexual and (d) queer. We grouped asexual or gray-A or demisexual and queer to a larger category called other sexual orientations and created two dummy variables (i.e., bisexual and other sexual orientations) with gay or lesbian serving as the reference group. These two dummy variables were later included in the mediation model as covariates.

### Data Analyses

All the analyses were conducted in Mplus 8.7. Preliminary analyses included descriptive and correlation analyses. Then, structural equation modeling was used to test the mediation model in which self-compassion and fear of compassion from others were examined as parallel mediators of the relationship between SGM-ACEs and psychological distress. Notably, psychological distress was a latent variable indicated by depression and anxiety. Covariates (i.e., age, gender, sexual orientation, educational level and income) were also included in the model predicting self-compassion, fear of compassion from others and psychological distress. Indirect effects were examined using the bootstrapping method with 10,000 bootstrap resamples (Shrout & Bolger, 2002). The difference between specific indirect effects for self-compassion and fear of compassion from others was also tested. Missing data were handled using full information maximum likelihood estimation (Enders, 2010). Model fit was determined by the following criteria (Hu & Bentler, 1999): (1) Standardized Root Mean Square Residual (SRMR) ≤ 0.08 (2) Root Mean Square Error of Approximation (RMSEA) ≤ 0.06 (3) Comparative Fit Index (CFI) and Tucker-Lewis Index (TLI) ≥ 0.95.

## Results

### Preliminary Results

Table 2 reports the frequency of exposure to SGM-ACEs in the current Hong Kong sample. The prevalence rates of exposure to different items of SGM-ACE among participants ranged from 3.8% to 62.8%. Across 8 items, 89.3% participants experienced at least one form of SGM-ACEs. Notably, prevalence rates varied significantly across different SGM-ACE items. Specifically, the most commonly reported adversities were vicarious trauma (i.e., witnessed or heard of physical harm to other SGM people, 62.8%) and regular exposure to transphobic, homophobic, or biphobic remarks from family members (61.5%). There are also substantial proportions of SGM individuals reporting bullying and discrimination experienced in faith community (44.2%) or school setting (30.3%), harsh familial treatment (41.3%) and internalized shame on SGM identities (26.9%). However, more severe situations were less commonly reported by SGM participants such as being kicked out of home or ran away (6.1%) or being institutionalized (3.8%) because of SGM identities.

Table 3 shows means, standard deviations and correlations of the study variables. SGM-ACEs were significantly correlated with higher depression and anxiety. SGM-ACEs were negatively correlated with self-compassion while positively associated with fear of compassion from others. Self-compassion was significantly correlated with lower depression and anxiety, whereas fear of compassion from others predicted higher depression and anxiety.

**Table 3:** Means, standard deviations, and correlation coefficients for variables

Variables	1	2	3	4	5
1. SGM-ACEs	-				
2. Self-compassion	− 0.25^**^	-			
3. Fear of compassion from others	0.22^***^	− 0.25^***^	-		
4. Depression	0.18^***^	− 0.34^***^	0.35^***^	-	
5. Anxiety	0.17^**^	− 0.34^***^	0.35^***^	0.71^***^	-
*M*	0.66	3.56	2.26	2.15	2.33
*SD*	0.70	0.58	0.75	1.62	1.68

^**^ *p* <.01, ^***^ *p* <.001

### Mediation Model Results

**Fig. 1:**
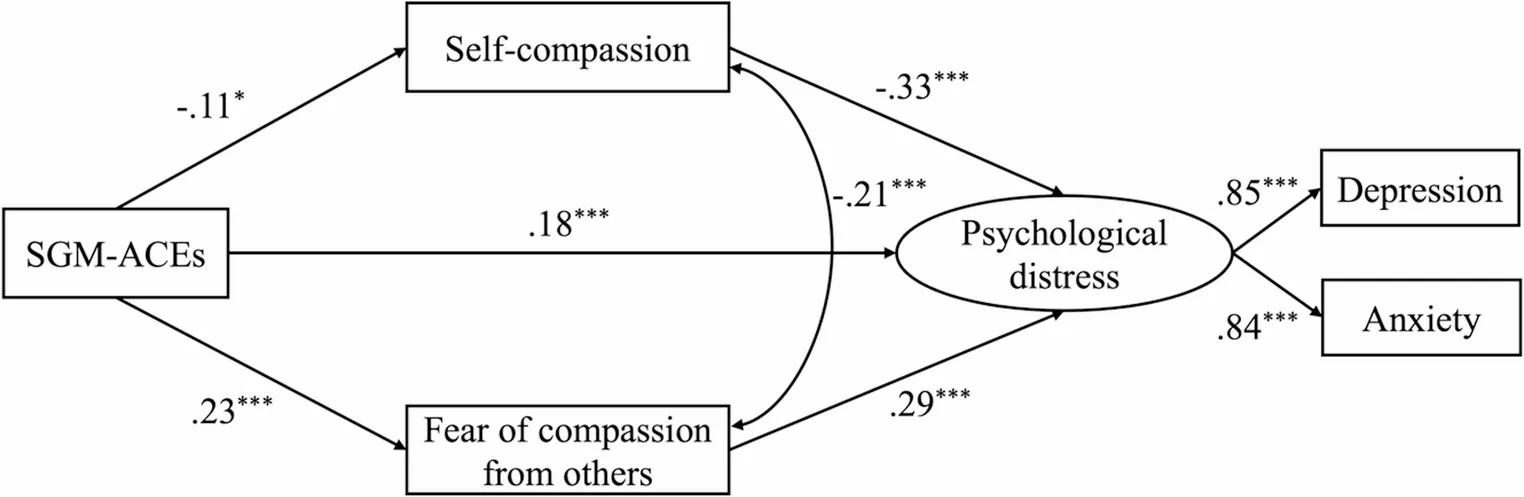
Mediation model results. *Note.* All path coefficients reported in the figure are standardized. Covariates (i.e., age, sexual orientation, gender, educational level and income) were included in the analysis but are omitted from the figure for parsimony. ^*^ *p* <.05 , ^***^ *p* <.001

The mediation model had an excellent model fit: CFI = 0.999, TLI = 0.998, SRMR = 0.007, RMSEA = 0.009, 90% CI [0.000, 0.045]. Figure 1 shows the results of the mediation model. As hypothesized, SGM-ACEs were significantly linked to higher psychological distress. Higher exposure to SGM-ACEs was associated with lower self-compassion and higher fear of compassion from others. Self-compassion was negatively linked with psychological distress while fear of compassion from others was related to higher psychological distress. We also correlated the residuals between self-compassion and fear of compassion in the mediation model and found a significant negative correlation between them. As for covariates, bisexual individuals reported lower levels of self-compassion compared with gay or lesbian people (*β* = *−.2*1, *p* <.05). Higher educational level predicted higher self-compassion (*β* = 0.14, *p* <.001). Age was negatively associated with fear of compassion from others (*β* = *−.0*9, *p* <.05). Higher income was significantly linked to lower psychological distress (*β* = *−.1*3, *p* <.01). From the bootstrapping results, both self-compassion (*B* = 0.07, *SE* = 0.03, 95% CI [0.01, 0.13], *β* = .04) and fear of compassion from others (*B* = 0.13, *SE* = 0.03, 95% CI [0.07, 0.21], *β* = .07) significantly mediated the relationship between SGM-ACEs and psychological distress when these two mediators were examined in the same model. However, the difference between indirect effects for self-compassion and fear of compassion from others was not significant (*B* = − 0.06, *SE* = 0.05, 95% CI [−0.16, 0.03]).

## Discussion

This study expanded extant ACE research among SGM people by utilizing the SGM-ACE measure, which assessed childhood trauma stemming from systemic oppression tied to SGM identities. The prevalence of SGM-ACEs and the association between SGM-ACE and psychological distress were examined in a non-US context among Hong Kong SGM individuals. In addition, the underlying mechanisms were explored by testing self-compassion and fear of compassion from others as two mediators. All the hypotheses were supported.

The present study extended SGM-ACE research to the Hong Kong context by examining the frequencies of SGM-ACEs and its psychological effects, which can provide valuable insights and empirical evidence for regions or countries with a comparable sociopolitical context as Hong Kong where LGBTQ-friendly policies and laws are still developing. Overall, the prevalence of SGM-ACEs was high among Hong Kong SGM individuals (89.3%). This high occurrence of SGM-ACEs may be attributed to cultural, social and legal barriers confronting SGM people in Hong Kong, where dominant discourses uphold traditional ideologies regarding sexual and gender diversity (Wong et al., 2025). Among different adversities, participants most frequently reported experiencing vicarious trauma from witnessing anti-SGM violence and regular anti-SGM remarks from family members. Notably, compared to a national sample of SGM adults in the U.S. (Schnarrs et al., 2023), our current sample appeared to report less frequent exposure to the list of adverse experiences measured. One speculated reason for this is the potential cross-cultural differences in behavioral expressions of anti-SGM attitudes. Items in the SGM-ACEs scale mainly capture overt forms of discrimination, such as encountering anti-SGM verbal comments, and being bullied or punished. While people in Hong Kong may hold negative attitudes toward SGM individuals, these may be expressed in a relatively covert and subtle way given the Chinese cultural notion of *hanxu* (含蓄), referring to a contained, reserved, implicit and indirect style of communicating feelings and thoughts (Kirkpatrick & Xu, 2012; Kwok & Kwok, 2022). For example, instead of explicitly saying anti-LGBT remarks, teachers in Hong Kong may avoid interacting with SGM students due to homophobia and transphobia (Hoang, 2019). However, the absence of discrimination does not equate the presence of safety and these less visible experiences can also induce harmful psychological effects (Diamond & Alley, 2022). Hence, it is pivotal for future studies to pay attention to these microaggressions experienced by SGM individuals in Hong Kong when studying their adverse childhood experiences. Nevertheless, the comparison should be interpreted with caution since the current sample may not be representative of the SGM population in Hong Kong.

Consistent with findings from U.S. samples, our results reveal that higher exposure to SGM-ACEs contributes to heightened psychological distress among SGM individuals in Hong Kong, demonstrating that the detrimental psychological effect of SGM-ACEs might be generalizable across different contexts. Results indicate that SGM individuals exposed to greater traumatic experiences due to their marginalized identities in childhood tend to exhibit lower levels of self-compassion and greater fear of receiving compassion from others in adulthood. This finding aligns with the notion from attachment theory that the internal working models determining how individuals relate to themselves and others are largely influenced by interactions with significant others in early life (Bowlby, 1973). Since SGM individuals with aversive childhood backgrounds often face homophobic family environments (e.g., being punished and shamed by family members), school bullying or community discrimination stemming from prejudice against their stigmatized identities, these adverse childhood experiences can interfere with their development of positive working models and secure attachment, disrupting the foundation for compassion (Mikulincer et al., 2005). Specifically, these SGM individuals tend to form a negative working model of themselves as unlovable and underserving of compassion (Chen et al., 2025). As such, they are less likely to recognize their needs for care and seek proximity or compassion from themselves or others in distressful times (Gilbert, 2009). Moreover, they may internalize the critical and harsh way that significant others treat them during childhood and endorse an uncompassionate and judgmental self-responding in adulthood (Mikulincer & Shaver, 2004). Additionally, these SGM individuals with aversive backgrounds tend to form a negative working model of others as rejecting, unreliable and even abusive (Chen et al., 2025). These internalized schemas may make SGM individuals fearful of depending on others in times of need due to the discomfort with closeness (avoidant attachment) or worry about rejection and abandonment (anxious attachment) (Collins, 1996; Diamond & Alley, 2022; Gilbert et al., 2011). SGM people may be inclined to these fearful and resistant reactions to compassion from others as a protective mechanism against anticipated victimization or rejection related to their stigmatized identities (Steindl et al., 2023).

The impaired ability to relate to oneself with compassion and the presence of fears and active resistance to receiving compassion from others were found to be in turn linked to higher psychological distress among SGM individuals, which is consistent with past findings (Carvalho & Guiomar, 2022; Gilbert et al., 2011; MacBeth & Gumley, 2012). Taken together, indirect effects were also consistent with expectations. Specifically, aligned with previous research findings illustrating self-compassion as an important mediating mechanism in the association between general ACEs and psychological outcomes (Guo et al., 2021; Kwok et al., 2022), this study revealed a significant mediation effect of self-compassion on the relationship between ACEs specific to SGM individuals and psychological distress. In addition, despite inadequate research attention paid to fear of compassion from others, the current study contributes to the existing research by identifying this construct as another significant mediator for the same link. Notably, the non-significant differences between indirect effects of self-compassion and fear of compassion highlight that both constructs play comparable, though distinct, mediating roles in explaining how SGM-ACEs affect psychological outcomes.

### Practical and Clinical Implications

The negative effects of SGM-ACEs on psychological distress and compassion-related psychological resources underscore the importance of diminishing the occurrence of SGM-ACEs through implementing interventions and practices across various social spheres, including families, schools, religious communities and societies.

Attachment-based family therapy and family-oriented trauma-focused cognitive behavioral therapy are key intervention approaches proposed to help parents cultivate mindsets and behaviors that support positive development among their SGM children within the broader cisheteronormative environment (Cohen & Ryan, 2021; Heiden-Rootes et al., 2025). These approaches guide parents in recognizing that certain well-intentioned behaviors, such as encouraging their children to conform to cisheterocentric norms, may inadvertently be experienced as rejecting or distressing, thereby heightening mental health risks and contributing to attachment insecurity that undermines access to compassion (Cohen & Ryan, 2021; Heiden‐Rootes et al., 2025; Ryan et al., 2009). Through these programs, parents can learn to engage affirmingly and responsively to the needs associated with their children’s SGM identities (Heiden‐Rootes et al., 2025; Ryan et al., 2010).

Additionally, as SGM students frequently experience bullying and discrimination related to their SGM identity in schools with hostile climates, school-based interventions and practices are essential. Implementing anti-bullying and anti-discrimination policies that specifically enumerate SGM students as a protected group can signal that discrimination and harassment based on SGM identity is prohibited (Russell et al., 2021). Furthermore, pre-service and in-service training for school administrators, educators, and staff on SGM-inclusive knowledge and skills is vital, as such training enhances awareness of homophobic bullying and equips personnel with concrete strategies to support SGM students (Kwok, 2019; Russell et al., 2021). Finally, school-based programs that foster empathy and social awareness may help students understand and appreciate diverse identities and perspectives, thereby reducing bullying behaviors and strengthening peer support for SGM students (Amadori et al., 2025).

The substantial prevalence of SGM-ACEs originating from faith communities highlights the need to enhance inclusiveness and affirmation for SGM individuals in religious settings. Efforts should focus on fostering both attitudinal and behavioral acceptance of SGM people within these communities. Considering the paramount role of clergy in shaping congregational beliefs, identifying religious leaders who support SGM rights could foster greater understanding and acceptance among community members (Gattis et al., 2014). Faith communities can also strengthen affirmation and through institutional practices, such as hosting annual celebration for SGM couples and recognizing openly SGM-identified clergy and members in the congregation (Lease et al., 2005).

Policymakers should not only enact policies such as the Mandatory Reporting of Child Abuse Ordinance but also allocate resources to training frontline workers and mandatory reporters so that they are equipped to respond appropriately to suspected abuse cases involving SGM children and youth while minimizing the risk of retraumatization within the child welfare system (McCormick, 2018; Salazar et al., 2023). For SGM youth involved in the juvenile justice system due to interpersonal conflict or engagement in punishable infractions, resources should similarly support training for educators, social service providers, and juvenile justice professionals to recognize biases and address discipline disparities faced by SGM youth (Poteat et al., 2016). These efforts should be accompanied by SGM-affirming healthcare services and public education to foster a more inclusive society.

Beyond reducing SGM-ACEs directly, the significant mediation results imply that therapeutic interventions targeting self-compassion and fear of compassion from others may serve as alternative and effective approaches for improving mental health outcomes of SGM individuals. Since the capacities for compassion are rooted in the attachment system, secure individuals with positive internal working models can be attuned to their own needs and able to provide compassion and affiliation for themselves (Bowlby, 1973). However, for individuals with histories of SGM-ACEs, attachment systems may become compromised as a result of early traumatic and abusive experiences (Gilbert et al., 2011). Therefore, it is imperative for therapists working with these individuals to attend to their clients’ attachment systems when facilitating them to cultivate self-compassion and mental well-being. Integrating findings and concepts from attachment research, compassion-focused therapy (CFT) might be particularly effective for enhancing self-compassion because it targets the social safeness system that evolves in parallel with the attachment system (Gilbert, 2005). This system functions as a soothing-contentment system, enabling individuals to respond to their distress and pain with self-calming and kindness (Gilbert, 2009). As this system is highly sensitive to interpersonal warmth, acceptance and care that are often absent among individuals exposed to SGM-ACEs, the crucial role of therapists is to serve as a “secure base” from which clients can safely explore their emotions, needs and thoughts during therapy (Gilbert, 2007). Consequently, clients’ social safeness system can be reactivated through attuned and caring therapeutic interactions. Additionally, central to CFT, compassionate mind training can effectively foster the inner warmth of individuals experienced SGM-ACEs by explicitly illustrating skills and attributes of compassion through specific activities such as compassionate imagery (Gilbert, 2009).

When it comes to addressing fears of compassion from others, therapists should first validate and de-shame clients’ fear and resistance to compassion and try to understand the function of fears (Steindl et al., 2023). For example, fear and resistance to compassion may play a protective role against rejection or hostility experienced by SGM people during childhood, functioning as a defense and coping mechanism (Steindl et al., 2023). Considering the negative psychological effects of fear of compassion from others, therapists can help clients recognize and understand the protective function of these fears while supporting them in developing embodied experiences of safeness through a trusting and warm therapeutic relationship (Steindl et al., 2023). Nevertheless, challenges may arise early in treatment because therapists’ displays of kindness and compassion can evoke grief associated with having sought, but not received, care from significant others (Gilbert et al., 2011). Some clients may become overwhelmed and dissociate to avoid the pain of this grief. Therapists should therefore validate these reactions and support them in processing these feelings (Gilbert, 2009).

Importantly, when working with SGM individuals, clinicians and therapists should ensure that their approaches are trauma-informed and LGBTQ+ affirming (Levenson et al., 2023; Liu et al., 2023). Trauma-informed care emphasizes that case conceptualization of SGM clients’ presenting problems and their therapeutic needs should be formulated within the context of their prior experiences of trauma and minority stress (Levenson et al., 2023). This can reduce stigmatization by shifting from a symptom-oriented perspective of “What’s wrong with you” to a curious and non-judgmental inquiry of “What happened to you” (Bloom, 2013). Therapists adopting a trauma-informed approach are expected to create a sanctuary for SGM clients, a safe space that validates all dimensions of identity (Bloom, 2013), while actively resisting re-traumatization by promoting an atmosphere of safety, trust, and empowerment to support resilience and healing for SGM individuals (Substance Abuse and Mental Health Services Administration, 2014).

### Limitations and Future Directions

First, although the strength of SGM-ACE scale lies in its capability to capture unique ACEs experienced by SGM people by accounting for cisheterosexism, this scale was initially developed using a regional sample in the U.S. (Schnarrs et al., 2023). Its applicability to other contexts such as Hong Kong remains uncertain. Further development is needed to capture early cisheterosexism experiences in diverse cultural contexts. Second, all measures included in the current study were self-reported, which may suffer from common method variance bias (Podsakoff et al., 2024). Future studies could address this issue by collecting data from multiple informants. Nevertheless, retrospective self-reports of ACEs have demonstrated sufficient validity for assessing such experiences (Hardt & Rutter, 2004). Third, the cross-sectional nature of the present study limits our ability to draw conclusions on the directionality or causality of different associations. Thus, longitudinal studies are warranted. Relatedly, we assumed all variables were static while they may actually fluctuate across contexts and situations. New insights can be generated by using an experience sampling design to examine how SGM-ACEs relate to the stability and volatility of momentary compassion-based processes and affective states. Fourth, since this study was based on a sample residing in Hong Kong society, whether similar effects of SGM-ACEs and the significant mediation effects of compassion could be generalized to other nations or regions still needs investigation, given varying levels of societal acceptance toward SGM individuals. Fifth, while this study mainly focused on the detrimental effects of childhood adversities and maladaptive psychological outcomes, it might also be crucial to explore resilience processes based on theories and scholarship. For example, chosen family or affirming relationships might mitigate the impact of SGM-ACE on compassion and in turn contribute to better mental well-being of SGM people (Greene & Britton, 2015; Huynh, 2023). Lastly, although this study contributes to the field by studying ACEs unique to the SGM population, it is important to acknowledge potential in-group differences among this population. For example, transgender and gender nonconforming individuals report greater adverse childhood experiences compared to their cisgender LGB counterparts (Schnarrs et al., 2019). Hence, future studies should investigate differences in frequencies and types of SGM-ACEs experienced by different SGM subgroups, and how SGM-ACEs may differentially relate to compassion or mental health outcomes across subgroups.

## Conclusion

Employing the SGM-ACE framework, this study investigated ACEs specifically related to SGM identities among SGM people in Hong Kong. Exposure to traumatic experiences due to SGM identities prior to adulthood was pervasive for SGM people in Hong Kong where SGM protections and policies remain underdeveloped. Consistent with previous studies conducted in the U.S., SGM-ACEs experienced during childhood significantly contributed to higher psychological distress in adulthood in the Hong Kong context. Furthermore, drawing from attachment theory, this study advanced the knowledge base by revealing both self-compassion and fear of compassion from others as significant mediators for explaining the relationship between SGM-ACEs and psychological distress. The findings underscore the importance of collaborative efforts among families, schools, religious communities and societies to create an inclusive environment for SGM individuals. The indirect effects highlight that therapeutic interventions addressing self-compassion and fear of receiving compassion from others may be beneficial for alleviating psychological distress of SGM people with identity-related ACEs.

## Data Availability

All study materials, data and code are available upon request.
